# Beta-Lactoglobulin as a Model Food Protein: How to Promote, Prevent, and Exploit Its Unfolding Processes

**DOI:** 10.3390/molecules27031131

**Published:** 2022-02-08

**Authors:** Alberto Barbiroli, Stefania Iametti, Francesco Bonomi

**Affiliations:** Department of Food, Environmental and Nutritional Sciences (DeFENS), Università degli Studi di Milano, Via G. Celoria 2, 20133 Milan, Italy; alberto.barbiroli@unimi.it (A.B.); stefania.iametti@unimi.it (S.I.)

**Keywords:** beta-lactoglobulin, unfolding intermediates, immunoreactivity, folding stability, ligands

## Abstract

Bovine milk beta-lactoglobulin (BLG) is a small whey protein that is a common ingredient in many foods. Many of the properties of BLG relevant to the food industry are related to its unfolding processes induced by physical or chemical treatments. Unfolding occurs through a number of individual steps, generating transient intermediates through reversible and irreversible modifications. The rate of formation of these intermediates and of their further evolution into different structures often dictates the outcome of a given process. This report addresses the main structural features of the BLG unfolding intermediates under conditions that may facilitate or impair their formation in response to chemical or physical denaturing agents. In consideration of the short lifespan of the transient species generated upon unfolding, this review also discusses how various methodological approaches may be adapted in exploring the process-dependent structural modifications of BLG from a kinetic and/or a thermodynamic standpoint. Some of the conceptual and methodological approaches presented and discussed in this review can provide hints for improving the understanding of transient conformers formation by proteins present in other food systems, as well as when other physical or chemical denaturing agents are acting on proteins much different from BLG in complex food systems.

## 1. Introduction

Beta-lactoglobulin from bovine milk is a small protein of 162 residues with a molecular mass of 18,281 Da (UniProtKB P02754) [1], and is present as a noncovalent homodimer in cow milk, a difference from the protein in milk from other species, such as mare or donkey. BLG is absent from human milk, and is a relevant allergen, in particular in early childhood. BLG belongs to the vast lipocalin family, and is thought to be involved in the transport of low-polarity micronutrients from mother to offspring [1].

This review focuses on the concept that controlled unfolding of proteins (and of other biopolymers, such as starch) is central to any process that implies the conversion of raw materials into foods suitable for human use from a number of standpoints: from accessibility of nutrients to removal of antinutritional factors, from inactivation of spoiling enzymes to microbiological safety, and including imparting desirable sensory traits to the processed food [2]. BLG has a number of molecular traits that make it well suited for addressing the nature, the rate of formation/decay, and the eventual fate of unfolding intermediates generated by processes of relevance to the food industry. In addition, the soluble nature of the protein and its relatively small size allow the use of a variety of approaches, which quite often may be integrated in a more comprehensive view of the occurring events.

BLG may be considered the epitome of a protein with diverse structo-functional regions, with a variety of interactions playing a role in its sensitivity to treatments of various natures [3]. In this respect, BLG has distinctly deformable regions in its structure. These regions involve a full complement of chemical features: from ionic interactions to covalent bonds, passing through hydrophobic interactions within the protein’s own regions and/or with micro- and macromolecular components of a given (food) system. As a consequence, BLG may undergo extremely varied structural modifications, involving both reversible and irreversible conformational changes [4,5,6].

The number of available three-dimensional native structures for the most diverse proteins is increasing exponentially [7], and also moving toward the study of huge complexes resolved by cryo-electron microscopy [8]. In contrast, studies on the transient conformations that a protein may assume in response to environmental stresses—such as those occurring in food processing—is still a challenge. To this end, techniques able to collect average signals from homogenous population of proteins in solution still represent a most advantageous choice, in particular for nonenzymatic food proteins such as BLG.

BLG is an excellent model for studies on individual determinants of process-induced conformational changes, as this protein offers the possibility of monitoring modifications in specific regions of the protein by exploiting different instrumental signals or by using a variety of methodological approaches. Fast, efficient, and almost inexpensive purification protocols, based mostly on salt precipitation [9] and chromatography [10,11], are available for BLG purification, making BLG a protein easily accessible from different sources. It is noteworthy that purification from raw milk should be considered mandatory whenever planning to use BLG for conformational studies, since the purity grade of commercial proteins is not extremely high (≈90%), and the presence of covalently linked dimers (and other polymeric species) has been reported [12].

The purpose of this review is to analyze current information on the features of unfolding intermediates of BLG, including conditions that may facilitate or impair its formation in response to chemical or physical denaturing agents. Please note that, for the sake of brevity and with the only exception of disulfide exchange events, this review will not consider modifications that result in covalent modification of specific amino acid side chains (such as the much studied process-dependent glycation [13] and the covalent attachment of fluorophores as relevant to cellular uptake or to structural studies), or of the primary structure (such as the release of bioactive peptides [14]).

Given the transient nature of the intermediates of the unfolding process and their short lifespan, it has been necessary to adapt methodologies that are fit to explore the dynamics of the process. In addition, the methodologies used in the studies considered here were adapted to conditions (such as the presence of other components) that could be of help in modeling the behavior of actual food systems.

Fluorescence, circular dichroism, and NMR are among the most useful and widespread methods for the structural analysis of proteins. Fluorescence exploits the intrinsic fluorescence of aromatic amino acids, with tryptophan providing the strongest signal. In the case of BLG, Trp19 at the calyx bottom is considered the best “reporter” of structural changes, with a sensible redshift of its emission maximum when it moves from the hydrophobic protein interior to a more hydrophilic environment. The other Trp residue in BLG—at position 61—is already solvent-exposed in the native structure of BLG. In addition, quenching of Trp fluorescence is useful to calculate the dissociation constant ligands either inserted in the central calix or bound on the surface [15,16]. Circular dichroism (CD) signals come from “intrinsic” chromophores, such as aromatic amino acids and disulfide bonds (relevant to tertiary structure, in the near-UV region) and peptide bonds (relevant to secondary structures, in the far-UV) [17]. Far-UV CD is rarely used for studies on BLG, as the protein is mainly structured in beta sheets that provide a weak signal only evident at very low wavelengths. On the contrary, BLG provides a characteristic and relatively strong CD signal in the near-UV region, stemming from 10 aromatic residues (2 Trp, 4 Tyr, 4 Phe) distributed quite uniformly throughout the sequence. A more analytical description of the structure can be achieved by ^1^H-NMR. At pH 2, where much of the BLG structure is preserved, two-dimensional spectra can be obtained to assign NH resonances, which can also be used in H/D exchange measurement [18,19].

In addition to the chromophores intrinsically present in the molecule, noncovalent binding of various spectroscopic probes (with distinct specificity) can be exploited. The widely used hydrophobic probe 1-anilino-8-naphthalene sulfonate (ANS) becomes fluorescent when bound to hydrophobic surface regions, and is commonly used to highlight the loss or gain of an exposed hydrophobic region during either transient or irreversible unfolding, even when no aromatic side chains are involved [20]. Moreover, displacement of ANS bound to specific regions of proteins may be used to assess competitive binding of other hydrophobic species [21]. Incidentally, it must be noted that BLG may also undergo covalent modification with a variety of fluorophores. Covalently labeled BLG species have been obtained by fluorescent derivatives targeting either cysteine or lysine side chains. These derivatives have been used in cellular uptake studies and may be of interest in setting up fluorescence resonance energy transfer (FRET) methods for structural studies of this protein. However, as stated above, methods involving covalent modification of BLG will not be considered here, for the sake of brevity.

BLG has a compact structure that makes its native conformation extremely resistant to proteolysis over a quite extended pH range [22]. Thus, sensitivity to proteases has been successfully applied as a tool to investigate the destabilization of the whole protein or of some specific region of its structure. When coupled with mass spectrometry for the exact identification of the released peptides, this approach makes it possible to identify the regions made accessible to individual proteases by conformational changes [23,24,25].

The same structural changes may also affect the accessibility of specific residues. BLG has five cysteine residues: four of them are involved in disulfide bonds, whereas the thiol moiety of the fifth one (Cys121) is hidden underneath the main alpha-helix, and therefore is inaccessible in the native protein. Thus, any movement of the C-terminus alpha-helix may be monitored by studying the reactivity of this thiol group toward specific reagents, such as Ellman’s reagent (5,5′-dithiobis-2-nitrobenzoic acid, DTNB) [26].

###  BLG Binding Properties

**Figure 1:**
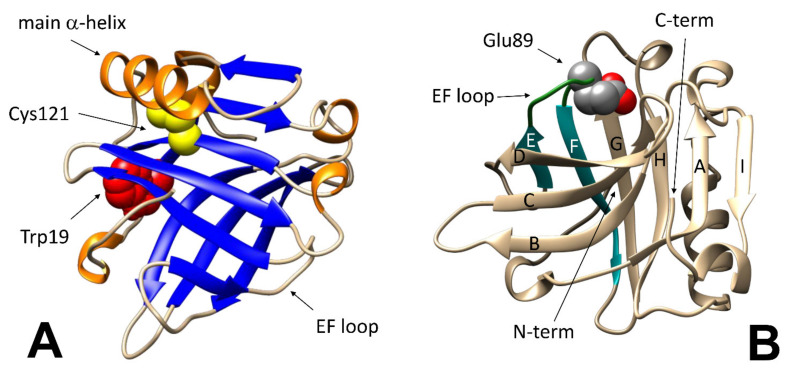
Features of the BLG native structure relevant to folding stability studies. (**A**): Cys121 (in yellow) is hidden under the main alpha-helix (in orange); Trp19 (red) is located at the bottom of the beta-barreled calyx (blue). (**B**): protonation of Glu89 (in CPK colors) triggers the closure of the “lid” formed by the EF loop (in dark green). Structures were generated by using the free graphical software UCSF Chimera (version 1.14, University of California, San Francisco, CA, USA).

A pictorial view of the regions relevant to the various methodological approaches discussed in this report is presented in the two panels of Figure 1, that highlights the fact that even modest changes in specific region may lead to easily detectable changes also when the overall geometry of the protein or its content in a given secondary structure element remains unchanged. As will be discussed later, local unfolding events may contribute to defining the threshold of reversibility for some of the most comprehensive structural changes as well.

Due to its peculiar characteristics, BLG has been used as model protein in many studies aimed at elucidating the biological implication of the protein carrier function. In native BLG, binding of ligands is known to involve different structural regions, depending on the physicochemical characteristic of the ligand [27]. Small compounds may be hosted in the central calix in competition with palmitate (the main natural ligand, which reportedly binds to the inner cavity of native BLG), stabilizing BLG toward chemical and physical denaturation. The bound molecule is protected and retains its biological activity even upon transient exposure to harsh chemical environments, such as those encountered during transit in the stomach. The compact structure of BLG at acidic pH and its relative insensitivity to pepsin have been hypothesized as being of great help in this regard [28]. Some compounds bind to sites that are physically distinct from the central calyx: between the main alpha-helix and strand G (involving Tyr102, Leu104, and Asp129), or in a groove between the first residues of strand B and the C-terminus (involving Trp19, Tyr20, Tyr42, Gln44, Gln59, Gln68, Leu156, Glu157, Glu158, and His161) [27]. They do not displace palmitate, and ternary adducts can be observed. This form of binding does not stabilize BLG against thermal denaturation. From a general point of view, the binding can be exploited to improve the solubility and/or the chemical stability of the ligand. Thus, BLG has been proposed as a delivery system for chemically sensitive or otherwise difficult-to-handle bioactives [28,29,30].

A number of approaches have been used to address the structural determinants of the binding ability toward compounds of great relevance from either a nutritional or a pharmacological standpoint, including polyphenols (which are dealt with in a dedicated subheading of this report). As detailed later on, in addition to spectroscopy, calorimetry, and other biophysical approaches, several studies based on bioinformatics modeling tools have appeared. However, all these studies addressed only very sporadically—if at all—the issues dealt with in the current review and concerning the impact of noncovalently bound ligands on the stability of the structure of BLG toward chemical and physical denaturation, as well as the rate of formation, the reactivity, and the lifespan of transiently/partially unfolded BLG molecules that may be formed in the process.

## 2. Destabilizing the BLG Structure

### 2.1. Chemical Destabilizing Agents

The sensitivity of BLG to changes in pH has been studied in great detail for decades. BLG is a dimer at neutral pH, but dissociates into monomers at low pH values, although the protein retains a compact overall structure (as demonstrated also by mono- and bidimensional ^1^H-NMR) at pH values as low as 2.0 [31]. Opposite charges on side chains in the C-terminus alpha-helices of facing monomers are relevant to noncovalent binding between monomers, and dimer stabilization also implies some sort of “leucine zipper” based on hydrophobic interactions among facing—and physically very close—aliphatic side chains in the long helix region in each monomer.

On a finer scale, the so-called “Tanford transition” is of particular interest, as it occurs at pH around neutrality, and affects the ability of the protein to interact with natural and synthetic ligands. In short, the protonation of the carboxyl group in the Glu89 side chain at pH below 6.0 allows the lid formed by the loop between strands E and F of the beta-barrel to close (Figure 1B), preventing access of ligands to the central hydrophobic cavity of BLG (or allowing to trap within the cavity molecules that may have accessed the protein interior at higher pH) [32].

Consistent with what was discussed above, both salts and nonionic chaotropes, such as urea, also may promote dissociation of the BLG dimer into monomers at pH values close to neutrality. Whereas the effects of lyotropic salts may just be due to the competitive screening of ionic interactions such as those involved in dimerization at neutral pH, the effects of lipotropic salts and of chaotropes are more extensive, and appear to involve regions of the protein other than the contacts between oppositely charged side chains reportedly involved in dimerization [33].

**Figure 2:**
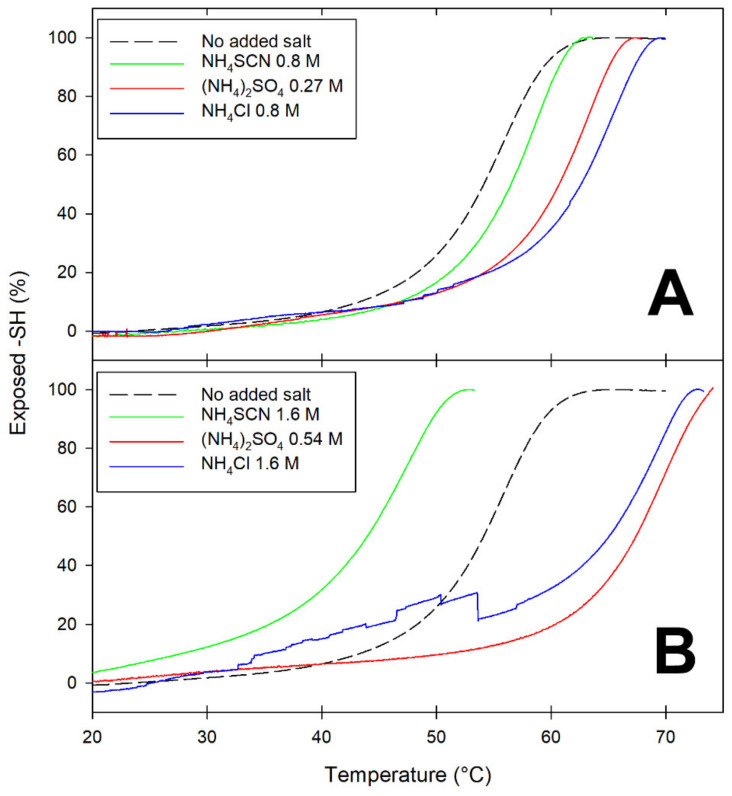
A comparison of the temperature dependence of the exposure of the Cys121 thiol as a function of the salt type and concentration. A BLG solution (1 mg/mL in 50 mM phosphate buffer, pH 6.8, containing 0.5 mM DTNB and the given salt concentrations) was progressively heated in a Peltier-controlled thermostated cell at a rate of 0.5 °C/min, with continuous monitoring of the absorbance of the solution at 412 nm. (**A**): salt concentrations corresponding to an ionic strength of 0.8 M; (**B**): salt concentrations corresponding to an ionic strength of 1.6 M.

Differences among salts at opposite ends of the Hofmeister series are exemplified by the data in Figure 2, which presents temperature-dependence studies on the accessibility of the Cys121 thiol as a function of the nature and concentration of added salts. As anticipated in the Introduction, in native BLG, this thiol is buried underneath the main C-terminus alpha-helix. Since movement of the alpha-helix away from the barreled body of BLG is known to be among the earliest and reversible steps of BLG unfolding [33,34], reactivity of Cys121 can be used to monitor this particular conformational change.

At ionic strength values below unity, all salts appear to have some stabilizing effect on BLG, as inferred by the increase in T_m_ (the temperature at which 50% of the thiols are exposed) (Figure 2A). At ionic strength around 1.6 M, the presence of lyophilic anions (sulfate and chloride) results in increased stability of BLG toward thermal denaturation: the more-solvated sulfate being more effective than the less-solvated chloride in increasing the temperature threshold for Cys121 exposure (Figure 2B). Conversely, high concentrations of the lipophilic anion thiocyanate (SCN^−^) have a devastating effect on the thermal stability of BLG, as indicated by the fact that 50% of the Cys121 thiol already becomes accessible at temperatures as low as 45 °C (Figure 2B).

Thus, a partially denatured form of a BLG monomer is present at physiological temperatures in the presence of high concentrations of the lipophilic anion thiocyanate. The “transient conformer” formed under these conditions was also found to undergo progressive polymerization, yielding well-structured fibrils that were not stabilized by disulfide bridges [24]. Noteworthily, much more ordered fibrils were obtained when thiocyanate was replaced by urea as the chaotrope used in fibril-formation studies. Intramolecular disulfide bonds play a major role in the formation and stabilization of the high-order polymers (regularly shaped twin beadstrings of submillimetric length) formed at subdenaturing urea concentration [21,35].

The properties of the “active monomer” generated by either chaotrope at subdenaturing concentrations and the mechanism leading to the formation of ordered polymers have been analyzed elsewhere [24], but it seems appropriate to underscore once again that the structural features of the “transient conformer” generated in the earliest steps of exposure to chemical denaturants dictate the rate and the fate of the subsequent (and much slower) formation of polymers of regular geometry.

### 2.2. Physical Destabilizing Agents

Temperature and high hydrostatic pressure (HP) are the physical agents most commonly used for food sanitation purposes. Both these treatments work by decreasing the structuring effects of solvent water toward hydrophobic contacts, which are of particular relevance in BLG. Even treatments of low intensity were found to result in structural modifications of the BLG structure, as made evident by spectroscopic measurements suggesting treatment-induced “swelling” of the protein structure. Below a given treatment threshold (typically, 10 min at 55–65 °C or at 400–600 mPa), the observed structural modifications were essentially reversible [4,20,36,37]. However, the transient conformers generated during the treatment were found to have some distinctive properties, including: (1) the ability to entrap suitable molecules when the BLG solution was cooled—or brought back to ambient pressure—and the protein returns to its compact native structure [38,39]; (2) a marked modification of their sensitivity to proteases, with the exposure of otherwise inaccessible hydrolytic sites and a substantially increased breakdown of the protein [25]. Both these events can be exploited for practical purposes.

As introduced above, partial unfolding of BLG (below the reversibility threshold) enhances the binding properties of BLG: relatively large bioactives may enter into the swollen protein structure, being “entrapped” when it is brought back to its compact native form. Trapping may occur either inside the calyx region of BLG, or on some hydrophobic ‘‘grooves’’ on its surface. The most evident of these ‘‘grooves’’ is located between the C-terminus helix and the “I” strand in the beta-barrel. The same region has been shown to be among the most temperature-sensitive in the structure of BLG [34]. In addition, the relevance of this region for trapping possible “payloads” after heating/cooling cycles has been elucidated by competition studies with palmitic acid [38]—which reportedly binds to the inner cavity of native BLG—and surface-bound hydrophobic spectroscopic probes.

Proteolysis of BLG conformers transiently formed during exposure of the protein to subdenaturing processes (i.e., below the reversibility threshold) has been exploited to target epitopes involved in the immunoreactivity and allergenicity of BLG [25,40]. It must be underscored that the extent of proteolysis required for removal of immunoreactive regions from transient intermediates is much lower than that required when dealing with native or irreversibly denatured BLG. In addition, physical removal of the proteolysis products obtained upon proteolysis of milk proteins under subdenaturing conditions yields large fragments (with decreased immunoreactivity) that may be directly processed into a quite large variety of dairy products. For these reasons, hydrolysis of “transient conformers” of BLG has been proposed [25] as an alternative to other processes (such as thermal insolubilization and extensive proteolysis) used alone or in combination for BLG removal in “humanized” bovine milk formulae.

**Figure 3:**
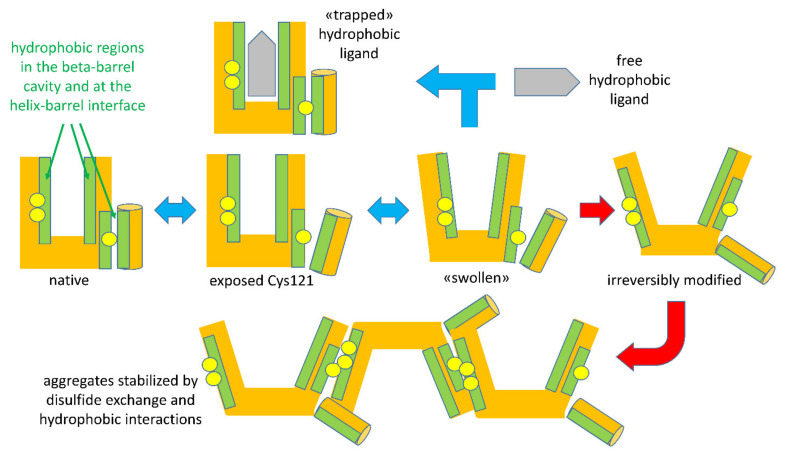
A schematic of the temperature-dependent denaturation events relevant to binding or entrapment and to formation of BLG aggregates. Reversible steps are indicated by blue double-ended arrows. Red arrows indicate irreversible steps occurring above a given temperature threshold (about 60 °C for ligand-free BLG).

When the intensity of physical treatments exceeds the reversibility threshold, structural modifications of BLG become irreversible. The irreversibility is prevalently due to a covalent modification of the protein structure: when a general unfolding involves the central beta-barrel, the disulfide bonds Cys66-Cys160 and Cys106-Cys119 became accessible to the reactive Cys121 of a second protein, triggering a thiols–disulfide exchange reaction (Figure 3). Among the two disulfide bonds, Cys residues of the surface-located Cys66-Cys160 bond were found to be more reactive compared to Cys106-Cys119 [41]. The extent of the reaction is related to the intensity of the thermal treatments, which affect the degree of exposition of disulfide bonds, as well the kinetics of the reaction [41]. This leads to the formation of polymeric forms of BLG, stabilized through hydrophobic interactions and—most relevantly—through disulfide exchange reactions involving the free Cys121 thiol, making acid-insoluble BLG aggregates a common (and legally recognized) marker of the intensity of milk thermal treatments.

Several studies have explored the dependence of the polymerization phenomenon (in terms of rate, mechanism, and yield), starting from very detailed studies on the influence of external factors such as the salt composition of the system, the pH, the protein concentration, and the presence of other species (see below for further details) that may affect individual steps of the unfolding/aggregation sequence, as does chemical modification of BLG through nonspecific glycation events. The practical relevance of all these points to the dairy industry (and to the food industry at large, given the widespread use of whey proteins as ingredients and the allergenic nature of BLG) may be taken as self-evident, and justifies the large number of reports in this particular area.

The highly schematic drawings in Figure 3 recapitulate the sequence of reversible and irreversible steps characterizing the unfolding of BLG by physical agents acting on the structure-forming properties of solvent water. Please note that the indicated events may undergo shifts in their onset threshold as a function of other components in the system, as will be discussed later.

### 2.3. BLG Denaturation at Interfaces

**Figure 4:**
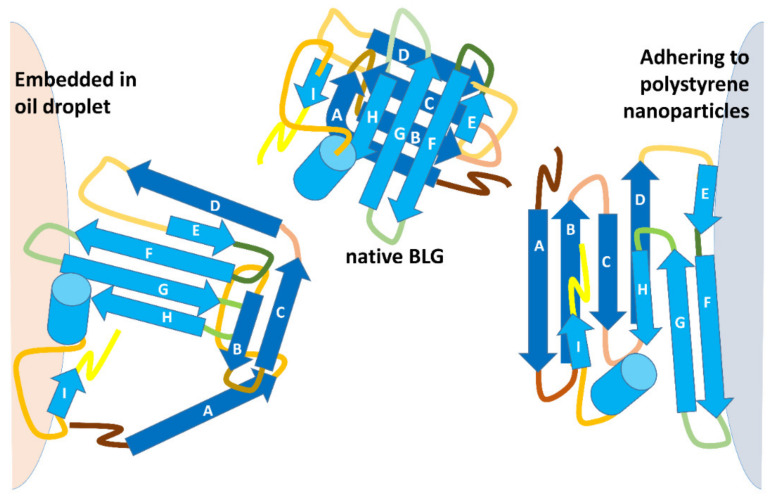
A schematic of the different types of BLG denaturation in oil–water emulsions and on the surface of hydrophobic NPs. The orientation of individual elements of the BLG structure was inferred from molecular dynamics and limited proteolysis studies [23,45].

Protein denaturation at interfaces plays a major role in food systems and in the food industry, and occurs upon contact between a protein and the interface formed between solvent water and hydrophobic components of the system. The latter may be oil droplets (as in emulsions), gases (as in foams) or solids (as in ice crystals in ice cream, dispersed solids such as those in coffee, or the metal surfaces in food-processing equipment). Stabilization of emulsions and foams typically calls for a previous or concomitant mechanical unfolding step, which may be accompanied (and modulated) by a thermal treatment [42] and pH control [43]. Aside from exposing hydrophobic sites on the protein, this combination of treatments also lowers the viscosity of fats and allows a decrease in the size of fat droplets with a concomitant increase in their surface area. As shown—very schematically—in the upper part of Figure 4, it would be expected that transiently exposed regions of BLG end up interacting with the hydrophobic interior of a fat droplet. Of course, as illustrated in Figure 4, penetration of the protein hydrophobic regions into the hydrophobic phase cannot take place in the case of hydrophobic solids, where different regions of the protein must come into play.

Studies on the molecular aspects of these events are far from abundant, and even less abundant are studies addressing the possible practical, nutritional, and health-related consequences of the different types of interaction of BLG with interfaces. The few available studies addressed not only structural changes in BLG, but also how these altered the pattern of action of digestive proteases and the immunoreactivity of the interface-adsorbed BLG, as well as of its digestion products.

Recent studies in this particular area have exploited the high surface area of small-sized (diameter of 20–200 nm) polystyrene nanoparticles as a model of hydrophobic surfaces for noncovalent BLG binding (the BLG dimer is about 2 nm across). Molecular dynamics studies indicated that unfolding of BLG on the styrene surface occurs at an extremely fast rate, being complete within 5 ns (that is, roughly 1000 times faster than unfolding in 8 M urea), generating products with altered immunoreactivity and with a very unusual pattern of trypsin sensitivity with respect to either native BLG or to otherwise denatured BLG. These effects were sensitive to the size of the nanoparticle, as if geometric or crowding effects were coming into play [23]. In this frame, it must be noted that covalent binding of BLG to the hydrophilic surface of dextran-coated magnetite nanoparticles of the same size had no effect on the immunoreactivity of the protein or on its ability to be recognized by monocytes, if not for enhancing the cellular uptake of the BLG-coated magnetic nanoparticles [44].

A comparison between the properties of BLG incorporated in emulsions and BLG stuck on the surface of polystyrene nanoparticles is provided in Table 1, which also offers a comparison with similar features in the native protein and of conformers originating from treatment with the different chaotropes (urea and thiocyanate) discussed above. In Table 1, the amplitude of the redshift of the Trp fluorescence emission maximum denotes changes in tertiary structure leading to increasing exposure of the Trp19 side chain to a hydrophilic environment, and appears similar for BLG at either solid/water or oil/water interfaces, but much lower than those observed in the presence of urea. Another difference worth underlining concerns the exposure of the Cys121 thiol, which is unaffected by BLG binding at the oil/water interface, but becomes similar to that of the temperature-modified protein when BLG is stuck on the surface of solid NPs.

As pointed out in previous studies [23,25,45], the most relevant difference (also in terms of practical significance to human nutrition) concerns the sensitivity of the various treatment-generated conformers of BLG to the action of proteases. Binding to interfaces (as well as temperature-induced unfolding) reportedly affects the extent and rate of proteolysis, also as a consequence of the difficulties that proteases encounter when recognizing potential proteolytic sites in the compact native structure of BLG.

**Figure 5:**
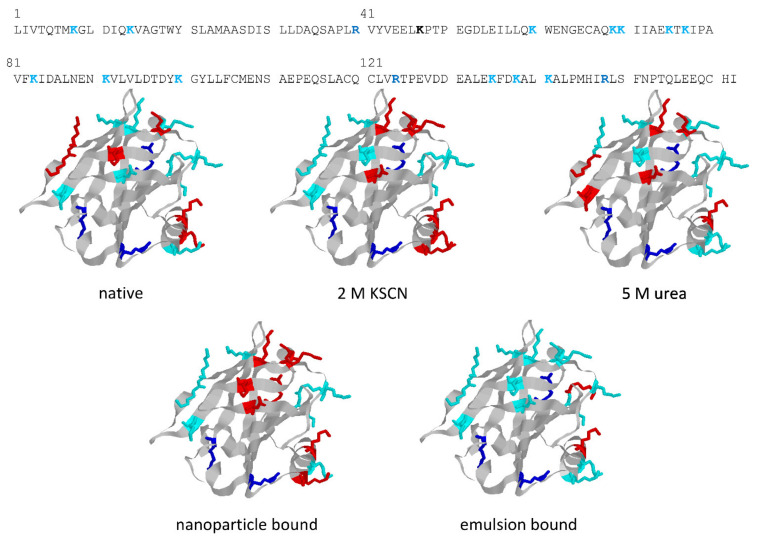
Action of trypsin on BLG conformers generated by different chemical or physical treatments. Sites of action of trypsin are highlighted in color (Lys, cyan; Arg, blue) in the sequence of BLG (top) and in the ribbon structures (all from the same view). Residues that are not accessible to trypsin hydrolysis upon protein unfolding in the given treatment conditions are in red. The sequence is from Uniprot (entry P02754), and crystallographic data are from PDB (entry 3UEU). Structures were built using the freeware Rasmol (rev. 2.7, www.OpenRasMol.org, accessed on 4 February 2022). Proteolysis data for the various conditions are from [23,24,45].

The structures in Figure 5 highlight the different trypsin sensitivity of conformers generated by various types of chaotropes and of the species adhering to polystyrene NPs or embedded into oil droplets in oil-in-water emulsions. Almost invariably, the residues made insensible to trypsin are in the C-terminus helix or on the opposite surface of the beta-barrel that outlines the central cavity of BLG, with only minor differences in terms of accessibility to trypsin. However, BLG bound at the oil–water interface (with concomitant mechanical unfolding) represents a remarkable exception, as all the lysine residues exposed on the barrel side opposed to the C-terminus helix are accessible to trypsin in this particular conformer.

These findings are of some interest, considering the allergenic nature of BLG (also related to its resistance to digestive proteases in the native form) and the widespread use of proteolysis as a way of lowering the allergenic potential of BLG. As also reported in Table 1, even extensive tryptic hydrolysis of the native protein has only a modest effect on its immunoreactivity. However, immunoreactivity decreases significantly when hydrolysis with trypsin is carried out on the conformers originating from exposure of the protein at temperatures at which reversibly modified BLG conformers are generated (55 °C in Table 1) [25]. Binding of BLG at either type of interface resulted in an increased immunoreactivity, a result that may be of practical relevance in consideration of the common use of BLG as a stabilizer in emulsions and suspensions. Quite surprisingly, the increased proteolytic sensitivity of interface-bound BLG did not result in a significant decrease in the immunoreactivity of the protein. This was attributed to the fact that the proteolytic products remain bound to the interface, and may be collectively recognized by the antibodies used in these studies [23,45].

### 2.4. Polyphenols: Stabilizing or Destabilizing Ligands?

In the last decade, many studies have focused on the noncovalent interaction between protein and food-derived ligands of both nutritional and physiological interest. Polyphenols took the lion’s share of these studies, also because of the ever-growing interest in their nutritional and functional and health-related properties. Most of the many reports in this area have dealt with the nutritionally relevant effect of the formation and stability of BLG/phenolics complexes, such the impact on digestibility and on immunogenicity.

Polyphenols from a number of sources have been found to bind on the external surface of the protein with binding constants in the 10^3^–10^5^ M^−1^ range, as calculated from the quenching of Trp19 fluorescence, and as also suggested by insilico docking studies. Computational approaches confirmed that flavonoids preferentially bind on the outer surface of BLG, mostly through hydrogen bonds and Van der Waals forces [16,46], and that the groove between the strand B and the C-terminus may represent a preferential binding site for several of these species [47]. At neutral pH, cocoa and coffee polyphenols were reported to induced structural destabilization of BLG, with a decrease in the β-sheet secondary structure in favor of α-helixes, and a concomitant increased susceptibility to pancreatin digestion. Conversely, all the polyphenolic extracts mentioned in these studies protected BLG from loss of structure at very acidic pH (around 1), slowing down pepsin digestion of the protein [10]. Since the binding site for polyphenols overlaps some of the epitopic regions in BLG [48], and considering that adsorption of polyphenols leads to a structural change in some regions of the protein, several of the BLG/phenolics complexes showed a decreased antigenicity [47].

To the best of our knowledge, the protective or destabilizing effects of polyphenols toward chemical or physical denaturation of BLG have been scarcely studied, and appears deserving further investigation. These studies also appear to be of particular practical relevance, in consideration of the sustainability concerns regarding the dairy sector at large and the use of dairy byproducts, as well as of ongoing research aimed at improving recovery of whey proteins and/or at promoting their use in non-food-related industries in the context of the so-called “circular economy”.

## 3. Stabilizing the BLG Structure

In what follows, this review will consider species that have a proven impact on stability of the BLG structure toward chaotropes and temperature, using two distinct classes of compounds as examples. One class is represented by osmolytes, such as sugars common in food formulations (e.g., sucrose), and by polyols, which are often used as stabilizers in industrial recovery of proteins, also from nonfood sources. Please note that reducing sugars (such as glucose and lactose) will not be discussed, in view in their role in a specific glycation of milk proteins (including BLG). A second class is represented by those ligands that are known to be noncovalently bound to “naturally occurring” BLG as isolated from milk in the absence of a specific removal step during protein isolation. Fatty acids are the most abundant species in this overall class, and the possible significance of their conditional binding to the behavior of the protein during processing of milk and milk derivatives has been seldom investigated, even if appearing to have potential practical interest.

### 3.1. Osmolytes

Organic osmolytes—such as sugar and polyols—are known to play an important role in stress protection by stabilizing macromolecules. Osmolytes act as protein stabilizer in view of their impact on their thermodynamic stability toward thermal [49] or chemical [50] denaturation. In the last century, the effect of osmolytes has been attributed to the so-called “preferential exclusion” [51], which implies that polyol/water and water/protein interactions are more favorable than polyol/protein interactions. Therefore, water molecules are “preferentially attracted” toward the surface of the protein, leading to the “exclusion” of polyols from the vicinity of the protein, resulting in a preferential hydration of the protein. From a thermodynamic point of view, osmolytes raise the midpoint of the apparent denaturation temperature (T_m_). Moreover, they are reported to have minor effect on the ΔG_D_° (free energy change at physiological condition) of the unfolding process. Thus, the native/unfolded equilibrium in the presence of osmolytes (but in the absence of denaturants) [52] and the native structure [53] are left practically unchanged. On the other hand, in the presence of a denaturant, the “thermodynamic” action of osmolytes is hypothesized to be mainly directed on the unfolded form of the protein increasing its energetic level, thus reducing the energetic gap between the native and the unfolded form that, in turn, disfavors the transition.

Although studies and theories regarding osmolytes are of long standing, in established laboratory practice glycerol is the one and only osmolyte used when working with “difficult” proteins, often with disappointing results. In this frame, a comparative investigation of the stabilizing properties of different osmolytes could be of practical relevance, as only a very limited number of studies of osmolytes on BLG has been published.

Trehalose, sucrose, and sorbitol were found to significantly increase the structural stability of BLG. Each of them leaves the native structure of BLG unaffected, and the effectiveness of their protecting effect was found to be: trehalose > sucrose ≅ sorbitol on a molar basis [53]. An in-depth characterization of the domain-specific differences shows that the differences among osmolytes are more pronounced in the case of the alpha-helix domain, whereas differences in the beta-barrel domain—which unfolds cooperatively after the alpha-helix—are least evident (Table 2). Differential scanning calorimetry (DSC) confirmed the entropic nature of stabilization by osmolytes in these regions, as well as in the central calix and the surface alpha-helix.

A molecular explanation of the observed behavior was provided by ANS titration experiments, which provided evidence of an increased affinity of ANS for surface hydrophobic patches in BLG in the presence of the largest osmolytes. Since osmolytes do not modify the native BLG structure, the increased “strength” of hydrophobic interactions in the presence of osmolytes (i.e., inside the preferentially excluded layer) was related to the ability of polyols to increase the surface tension of water, acting as “tension-inactive” substances. In other words, inside the preferentially excluded layer, the hydrophobic/hydrophilic contacts are further disadvantaged. As explained above, from a thermodynamic point of view, the exposure of hydrophobic regions to water upon denaturation leads to a higher energetic level of the systems, thus reducing the energetic gap between the native and the unfolded forms [53]. Glycerol was found to be less effective than its “big brothers” sorbitol, sucrose, and trehalose. Interestingly, glycerol has no effect on the affinity of BLG for ANS, suggesting a different mechanism of action, which probably excludes preferential exclusion [53]. Noteworthily, differences between the various polyols discussed above are much less evident if the concentration dependence of their effects is estimated on a weight basis, suggesting that their activity could be related to other molecular characteristic, (e.g., the number of oxydrilic groups able to interact with water).

Apart from the interest in the theoretical modeling of the phenomenon, studies on the “protective” effects of cosolutes acting on the water–protein interaction are of practical relevance in food systems. Indeed, the involved domains have a key role in BLG, and are relevant—among others—to the protein’s ability to form a stable polymeric network when suitably concentrated BLG solutions are heated at appropriate pH values above a given temperature threshold.

As discussed above and as shown schematically in Figure 3, formation of these polymeric species involves noncovalent contacts among hydrophobic regions, and may lead to covalent binding through thiol–disulfide exchange reactions triggered by the independent movement of the helix domain shielding the Cys121 thiol from intermolecular contacts with cysteine disulfides in other structural regions of BLG [4,5].

### 3.2. Stabilizing Ligands

Like all other proteins belonging to the lipocalin superfamily [55], BLG binds hydrophobic ligands. Some of the small-sized ligands bind to the central hydrophobic calyx [18], but others have been reported to bind to hydrophobic “grooves” on the protein surface [47,56,57]. Typically, calyx-bound ligands having an “elongated” structure (such as retinol or fatty acids) make the protein less sensitive to chemical and physical denaturation, whereas surface-bound ligands (including the phenolics discussed above) only marginally affect the sensitivity to denaturants of the overall BLG structure [37]. Native BLG has been demonstrated to bind different compounds, such as fatty acids [54,58], compounds of pharmaceutical relevance [28], retinol [59], vitamin D [60], and polyphenols [16]. Palmitic acid is the most frequent natural ligand bound in the central-calyx of BLG in bovine milk [54]. Binding of palmitate requires the “lid” (i.e., the EF loop) to be in the open form, which is typically present at neutral pH. Fatty acids bind to the calix/barrel of BLG with their methyl end deeply buried within the protein and the carboxyl group close to the “open end”, with hydrogen bonding to Glu62 and Lys69 [61]. At pH lower than 6.0, protonation of Glu89 triggers the movement of the EF loop, so that the “lid” closes access to the central calix [62] (Figure 1), with concomitant release of palmitic acid [18].

Binding of palmitic acid at pH around neutrality—as in milk and in sweet whey—stabilizes the protein against physical denaturants and chaotropes [58]. As shown in Table 2, midtransition parameters increase markedly in the presence of palmitic acid: from 72 to 81 °C in thermal denaturation, and from 4.0 to 4.8 M urea in chemical denaturation, as monitored by near-UV CD and Trp fluorescence, respectively. Interestingly, although the ligand is only contacting the inner side of the calix, the domain involving the surface alpha-helix also is stabilized. Indeed, the time course of exposition of reactive Cys121 is sensibly slower in palmitate-saturated BLG than in the ligand-free protein [54]. The calyx-bound palmitate thus makes the movement of the helix region that exposes Cys121 during the earliest steps of unfolding more difficult. This could imply that palmitate bound to the calyx interior acts on some structural “latch” that stabilizes the closed position of the helix from the outside of the calyx itself.

For the sake of comparison, two ligands of pharmacological interest have also been included in Table 2 as representing the two different ways of BLG–ligand interactions, as assessed through ^19^F-NMR spectroscopy [28]. Fluvastatin, which binds in the central calyx of BLG in the same manner as fatty acids and retinol [18,38,61], shows stabilizing effects comparable to those of palmitic acid. Conversely, flurbiprofen, which binds to hydrophobic regions on the protein surface in the same manner as most polyphenols and other hydrophobes that contain aromatic rings (such as ANS) [4,20,21,38,56], has no effect on BLG stability as measured through spectroscopic approaches [28].

## 4. Conclusions and Perspectives

The collection of studies presented in this contribution highlights some of the significant progresses made in the understanding of the relevance of unfolding intermediates and of factors governing the rate of their formation and of their subsequent conversion in food proteins. It should be underscored that the overall concept of addressing the structural features of a “transient” unfolding intermediate may be seen as a change in perspective in studies on food proteins. In this general area, for a number of reasons, a number of excellent studies have addressed the impact of sometimes subtle changes of the protein profile in individual starting materials, as well as the effects on protein features (from a chemical, structural, or biological and nutritional standpoint) that only became evident in the finished product. While of the highest practical interest, these studies may sometimes come up short of the predictive value, which can only be inferred when unveiling the molecular properties and the rates of formation or decay of protein conformers that very often dictate the outcome of a given process and the overall quality of the product.

Admittedly, extending these concepts to systems other than solutions of a globular protein with all the easy-to-monitor features discussed here in the case of BLG may lead to pretty slippery grounds. However, some of the conceptual and methodological approaches discussed in this review are now being tested for use in other food systems, including a low-water system containing insoluble and/or highly polymeric proteins, along with a plethora of micro- and macromolecular potential interactors.

Some applications of these concepts can be already found in the literature, and range from gluten-based or gluten-free pasta/bakery products to legume-based or egg-containing foods. In several cases, molecular-based approaches related to what we presented here have been used in combination with physical measurements, such as various rheology-based approaches, with mutual benefit for the food technologist and the protein biochemist. In conclusion, it seems safe to forecast that extending these collaborative approaches to other actors in the food-system scene (from crop/animal scientists to experts in microstructured materials or in human nutrition) will make the “science for food” concept brought forward in this review both permanent and pervasive.

## Figures and Tables

**Table 1:** Features of the BLG conformers generated by physical and chemical denaturation or by adhesion to interfaces.

Measured Parameter	Treatment or Protein Status					
	Native, No Treatment	55 °C	2 M KSCN	4 M Urea	Stuck onto Polystyrene NPs	Embedded in Oil Droplets
Maximum emission of Trp fluorescence, nm	338	344	338	355	344	345
Reactivity of the –SH group in Cys121	Very low	High	Low	Very high	High	Low
Novel or unexpected trypsin- resistant sequences	-	41–70-(S-S)-149–162	1–45 113–124-(S-S)-149–162	1–45 142–162	139–148 61–70-(S-S)-102–162	61–105 61–69-(S-S)-149–162
Relative immunoreactivity, intact protein ^1^	100	n.a.	n.a.	n.a.	300	200
Relative immunoreactivity after tryptic hydrolysis	30	<5 ^2^ 20 ^3^	n.a.	n.a.	300	180

^1^ Measured with a monoclonal antibody (5G6, courtesy of Hanne Frokiaer, University of Copenhagen) [23,45]; ^2^ measured with the 5G6 monoclonal antibody on large-size hydrolysis fragments [25]; ^3^ measured with a polyclonal antibody on the unresolved hydrolysis products [25].

**Table 2:** Denaturation midpoints (temperature or chaotrope concentrations) for BLG in the presence/absence of ligands and of other components in the system.

	Tm (°C)		Cm (Urea, M)	Note
BLG, no additions		72.0 ^1^	4.5 ^1^	Near-UV CD
			4.0 ^1^	Intrinsic fluorescence
	55.0 ^1,2^	70.5 ^1,2^		DSC
** Ligand **				
Palmitate		81.0 ^1^	5.7 ^1^	Near-UV CD
			4.8 ^1^	Intrinsic fluorescence
	63.0 ^1^	83.5 ^1^		DSC
Flurbiprofen		71.0 ^3^		Near-UV CD
Fluvastatin			5.65 ^3^	Near-UV CD
** Osmolytes **				
Glycerol	54.6 ^2^			Thiol accessibility
		74.0 ^2^		Near-UV CD
Sorbitol	58.3 ^2^			Thiol accessibility
		80.0^2^		Near-UV CD
Sucrose	60.1 ^2^			Thiol accessibility
		81.5 ^2^		Near-UV CD
Trehalose	64.0 ^2^			Thiol accessibility
		79.6 ^2^		Near-UV CD

^1^ From [54]; ^2^ from [53]; ^3^ from [28].
